# Performance of PATC-PDMDAAC Composite Coagulants in Low-Temperature and Low-Turbidity Water Treatment

**DOI:** 10.3390/ma12172824

**Published:** 2019-09-02

**Authors:** Peng Zhang, Lina Liao, Guocheng Zhu

**Affiliations:** 1 College of Environmental Science and Engineering, Taiyuan University of Technology, Taiyuan 030024, China; 2 College of Civil Engineering, Hunan University of Science and Technology, Xiangtan 411201, China

**Keywords:** aluminium chloride, titanium tetrachloride, poly(diallyldimethylammonium chloride), composite flocculant, low temperature, low turbidity

## Abstract

A novel composite was synthesized by using flocculant polyaluminum titanium silicate chloride (PATC) and poly(diallyldimethylammonium chloride) (PDMDAAC) monomers to treat low-temperature and low-turbidity water. The structure and physicochemical properties of PATC-PDMDAAC were analyzed by Fourier transform infrared spectroscopy (FTIR), thermogravimetric analysis/differential scanning calorimetry (TG/DSC), X-ray diffraction spectroscopy (XRD), and scanning electron microscopy–energy dispersion spectrum (SEM-EDS). The compound flocculant produced new functional groups exhibiting great thermal stability, and the complex chemical reaction between the two monomers generated new substances with reticular structures. Coagulation performance results showed that the PATC-PDMDAAC had an organic and inorganic ratio of 0.15 and exhibited excellent removal efficiency at pH 9.0, dosage of 1.80 mg/L, sedimentation time of 40 min, and a stirring speed of 110 r/min. The optimal treatment efficiency reduced the turbidity to 0.56 NTU (Nephelometric Turbidity Unit). The removal rates of TOC (Total Organic Carbon) and UV254 (Ultraviolet 254) were 62.18% (from 7.23 mg/L to 2.734 mg/L) and 99.99% (from 10 mg/L to 0.001 mg/L). The 3D fluorescence, zeta potential and kinetic analysis in the flocculation process indicated that coagulant electroneutralization and adsorption bridge in a slightly alkaline environment played a dominant role, and a sufficient and effective collision occurred between the coagulant and particulate matter under the optimal dosage. Lastly, PATC-PDMDAAC has more advantage than conventional flocculants in the treatment of low-temperature and low-turbidity water in the Xiangjiang River.

## 1. Introduction

Low-temperature and low-turbidity water adversely affects coagulation and precipitation owing to its chemistry and dynamics. The rate of coagulation reaction in such water is slow, and the flocs that subsequently form are small, light, loose, and difficult to efficiently precipitate. Several reasons may explain these phenomena. One of the reasons is the slow Brownian motion due to the increase in the inertia of impurity particles at low temperature. The other reason is the increase in viscosity and subsequent increase in resistance between liquid layers; the enhanced resistance limits the collision between particles [1]. In low turbidity water, the number of impurity particles is small, and the distribution is relatively uniform. Colloidal particles have small negative potential and strong interparticle rejection. Accordingly, colloids are difficult to remove, and the aggregation effect is poor [2]. Therefore, improving the purification efficiency of low-temperature and low-turbidity water is a great challenge.

In an aluminum coagulant, which is a kind of flocculant commonly used in the coagulation process, hydrolyzed Al hydrates with water molecules to form positively-charged metal ions; simultaneously, aluminum ions combine with ionized OH^−^ in the aqueous solution to generate positive ions, such as Al(OH)^2+^, Al(OH)_2_^+^, and Al(OH)_3_; these products can electroneutralize the negative colloid in the solution, reduce the zeta potential (ZP) of the pollutant, and make the stable colloid unstable [3]. Moreover, the products can promote different degrees of polymerization through the bridging action of –OH to generate multicore complex ions and [Al(OH)_3_]m(s). These polynucleated complex ions have long molecular chains, and many adsorption sites can adsorb pollutant particles in water. Such ions can intercept and precipitate pollutants in water through adsorption bridging [4,5]. Therefore, aluminum coagulants remove colloid particles and other pollutants in water mainly through the electroneutralization effect of metal hydrolysis and the adsorption bridging effect of macromolecular complex ions formed by hydrolysis polymerization. However, the effect is poor in the coagulation reaction process when aluminum salt is added to low-temperature and low-turbidity water. This outcome is attributed to the aluminum salt coagulant, which is endothermic when hydrolyzed. However, the rate of hydrolysis is slow at low water temperatures. Therefore, increasing the hydrolysis rate of aluminum salt is helpful in improving the treatment efficiency of low-temperature and low-turbidity water at low temperature.

Titanium salt coagulant has excellent turbidity removal and decolorization effect and is safe and nontoxic to the human body. In 1937, titanium sulfate was first used in water treatment by Upton and Buswell [6], and it was found that the coagulation effect was excellent, titanium sulfate exhibits not only higher removal efficiency for chromaticity but also a faster hydrolysis rate and better coagulation effect at low temperature than aluminum salt. It was found that titanium salt flocculants have better effluent quality, wider range of pH adaptability, and better flocculation performance than traditional chemical flocculants according to Lokshin and Belikov [7]. Titanium salt has a good coagulation effect at low temperature and thus suitable for water treatment in winter. According to Huang et al. [8], polysilicate titanium was prepared by mixing polysilicate acid and titanium sulfate solution and used it in the treatment of simulated wastewater of humic acid-kaolin; the compound had a good removal effect on turbidity and natural organic matter (NOM). The introduction of titanium ions into aluminum coagulants not only can replace part of aluminum ions to play a flocculation role but can also hydrolyze and polymerize titanium salts into multicore polymer products at a low pH; accordingly, the degree of polymerization and molecular weight of coagulants and the coagulation effect are enhanced [9]. Therefore, the introduction of titanium ions into traditional aluminum coagulants will improve the hydrolysis rate and coagulation effect of aluminum salts.

The influence of water temperature is reduced when a polymer electrolyte is used as coagulant or inorganic and organic coagulant compounds are prepared in the treatment of low-temperature and low-turbidity water. The turbidity removal rate of low-temperature and low-turbidity water can exceed 90% when polyaluminum chloride, modified activated silicic acid, and polyacrylamide solutions are simultaneously used, the turbidity removal rate can be less than 80% when polyaluminum chloride was used alone as shown by Xu et al. [10]. According to Lou et al. [11], polymer-polyferric chloride (PAFC) and its combination (FeCl_3_/PAFC) were used to treat low-temperature low-turbidity water; the results showed that the turbidity of effluent can be reduced to less than 0.5 NTU (Nephelometric Turbidity Unit) after the combination of two conventional coagulants. The feasibility of treating low temperature low turbidity water with high-basicity polychloride and high-viscosity chitosan was studied by Zhang et al. [12], the result showed that the compound of high-basicity PAC (Poly Aluminium Chloride) (90.3%) and high-viscosity chitosan (500 m·Pas) and the turbidity removal rate reached 87%. Poly(diallyldimethylammonium chloride) (PDMDAAC), a commonly used organic coagulant in waterworks, exhibits several advantages, such as large molecular weight, high positive charge density, nontoxicity, and strong ability to adsorb and bridge colloidal substances with negative charge in water [12,13].

In this study, inorganic coagulant polyaluminum titanium silicate chloride (PATC) prepared in laboratory and organic coagulant polydimethyl diallyl ammonium chloride were used as raw materials for the synthesis of inorganic-organic composite flocculant PATC-PDMDAAC. The optimal treatment conditions at low temperature and turbidity were determined by a single variable method. The mechanism of PATC-PDMDAAC in the treatment of low-temperature and low-turbidity water was analyzed with 3D fluorescence, zeta potential, and coagulation kinetics. The effects of the prepared coagulant and a conventional coagulant in the treatment of low-temperature and low-turbidity water in the Xiangjiang River were compared.

## 2. Materials and Methods

### 2.1. Materials

PATC used in this study was prepared in our laboratory. All reagents used in this study except Titanium tetrachloride (TiCl_4_) were of analytical. TiCl_4_ was chemically pure which was provided by Damao Chemical Reagent Industrial company (Tianjin, China). Aluminum chloride (AlCl_3_) was purchased from Tianjin Hengxing Chemical reagent company (Tianjin, China). Sodium silicate (Na_2_SiO_3_) was sourced from Hunan Huihong Reagent Co., Ltd. (Changsha, China). Sodium hydroxide (NaOH) was obtained from Xilong Scientific Co., Ltd. (Shantou, China). Concentrated sulfuric acid (H_2_SO_4_) and Hydrochloric acid (HCl) were from Zhuzhou Starry Glass Co., Ltd. (Zhuzhou, China). The kaolin was purchased from Damao Chemical Reagent Industrial company (Tianjin, China). Humic acid (HA) and Poly (diallyldimethylammonium chloride) solution (PDMDAAC, 20 wt%) were purchased from Aladdin Industrial Corporation. All aqueous solutions were prepared with ultrapure water using an ultrapure LBY-20 water purifier which was provided by Chongqing OWEN Science and Technology Co., Ltd., (Chongqing, China). The electronic analytical balance was purchased from Shanghai Hengping Scientific Instrument Co., Ltd. (Shanghai, China) used to weigh drugs. The PB-10 acidity meter used to adjust the pH was come from Sartorius Scientific Instrument (Beijing, China) Co., Ltd. (Beijing, China). The ZR4-6 program-controlled jar test apparatus used for coagulation was obtained from Zhongrun Water Industry Technology Development Co., Ltd. (Shenzhen, China). The heat-collecting magnetic stirrer was supplied by Bonsai Instrument Technology (Shanghai, China) Co., Ltd. (Shanghai, China). The 2100Q turbidimeter was produced by HACH (Loveland, CO, USA). The Nicolet 6700 FTIR was sourced from Thermo Fisher Scientific Company (Waltham, MA, USA). The 8D-Advance XRD was purchased from Germany (Karlsruhe, Germany). The STA 409 PC/4/H thermal analyzer came from Naichi Instrument Manufacturing Co., Ltd. (Karlsruhe, Germany). The SU-5000 SEM was obtained from JEOL (Tokyo, Japan). The ultraviolet-visible spectrophotometer (twin-beam type TU-1901) was produced by Beijing Purkinje General Instrument (Beijing, China). Co., Ltd. The Vairo TOC was supplied by Elementar Company (Frankfurt, Germany). The zetasizer 2000 was obtained from Malvern (Malvern, UK). The F-4600 fluorescence spectrophotometer was purchased from Hitachi High-Technologies Corporation (Hitachi, Tokyo, Japan).

### 2.2. Preparation of PATC-PDMDAAC

PATC was prepared by using AlCl_3_, Na_2_SiO_3_, and TiCl_4_. First, 0.5 mol/L sodium silicate solution was configured, then the pH of the solution was adjusted with concentrated sulfuric acid until the pH was 1.95 ± 0.05. Polysodium silicate solution (solution A) was obtained by magnetic stirring for 60 min at room temperature. Subsequently, 0.01 mol AlCl_3_ was dissolved in a deionized water solution and then frozen for 30 min until crystals appeared. Thereafter, the TiCl_4_ was added to the AlCl_3_ solution with crystals. Solution B was obtained after the NaOH was completely dissolved in the mixed aqueous solution under continuous stirring at room temperature, and stirring was continued in the water bath for 60 min. The products obtained from solutions A and B were mixed with magnetic force for 60 min under certain conditions. PATC was obtained by curing for 24 h.

PDMDAAC was injected into the prepared PATC solution under continuous stirring for 120 min. The reaction temperature was 50 °C, and the PATC-PDMDAAC was obtained by curing for 24 h.

### 2.3. Characterization of the Flocculant

In this section, the chemical structure and apparent morphology of PATC-PDMDAAC series samples were compared and analyzed. The liquid PATC-PDMDAAC was put into 60 °C drum wind drying oven, and then dried and grinded into powder. FTIR spectra of PATC-PDMDAAC, PATC, and PDMDAAC were recorded using KBr pellets through a Nicolet 6700 FTIR (Waltham, MA, USA), and the tested wave numbers was set between 400 and 4000 cm^−1^. The specific characteristics of the samples were obtained from D8 ADVANCE XRD and then scanned and analyzed with Jade 6 software (Livermore, CA, USA). The scanning angle control was 5°–90°. The surface morphology of PATC and PATC-PDMDAAC was observed using a SU-5000 SEM (Tokyo, Japan), and the specific element analyses of PATC and PATC-PDMDAAC were carried out via EDS (Tokyo, Japan). The STA 409 PC/4/H thermal analyzer (Naichi, Germany) was used to identify and quantitatively analyze the chemical composition of substances, while investigating the thermal stability of PATC-PDMDAAC with different proportions. The thermal gravimetric analysis (TG) was carried out at a heating rate of 10 °C·min^−1^, temperature range of 30–900 °C, and a nitrogen flow of 10 mL·min^−1^. The Al–ferron complex timed colorimetric method was used to determine the Al speciation of PATC-PDMDAAC with different material ratios, the absorbance value of the Al–ferron solution was determined at a 362 nm wavelength using the UV–VIS spectrophotometer (twin-beam type TU-1901, Beijing Purkinje General Instrument (Beijing, China). Co., Ltd., Beijing, China).

### 2.4. Performance

The flocculation efficiency of PATC-PDMDAAC was evaluated for the treatment of low-temperature and low-turbidity water, which was prepared with humic acid and kaolin suspension. The humic acid-kaolin wastewater was prepared as follows: 1.000 g of humic acid (HA) and 0.040 g of NaOH powder were dissolved in 800 mL of deionized water with mechanical stirring for 60 min. When the humic acid powder was completely dissolved, the volume was fixed to 1000 mL, and the humic acid reserve solution with a concentration of 1000 mg/L was obtained. Predried 5.000 g of kaolin power was dissolved in 1000 mL of deionized water with stirring for 60 min. The supernatant (500 mL) was removed by siphon method after standing for 30 min. The humic acid-kaolin simulated wastewater was obtained by diluting 10 mL of humic acid reserve solution with deionized water to a constant volume of 1000 mL and adjusting the turbidity with kaolin reserve solution. The simulated water concentration of HA was 10.0 mg/L, and kaolin suspension was used in the adjustment of the initial turbidity of the simulated water sample. The characteristics of simulated and natural water (Xiangjiang River) are shown in Table 1.

The flocculation experiment was conducted on a ZR4-6 program-controlled jar test apparatus (ZhongRun Water Industry Technology Development Co., Ltd., Shenzhen, China). A certain amount of PDMDAAC-PATC (based on PATC) was added to the simulated water sample. It was first stirred at 210 r/min for 2 min, then stirred at 50 r/min at a slow speed for 10 min, and finally settled for half an hour. After flocculation process, the supernatant at the depth 2 cm below the liquid surface was extracted and its residual turbidity, UV254 (Ultraviolet 254) and TOC (Total Organic Carbon) were detected. The zeta potential was measured through a zetasizer 2000 (Malvern, UK). Furthermore, the residual HA was detected by F-4600 three-dimensional fluorescence (Hitachi High-Technologies Corporation, Hitachi, Tokyo, Japan).

## 3. Results and Discussion

### 3.1. Characterization

#### 3.1.1. FTIR Spectra Analysis

**Figure 1:**
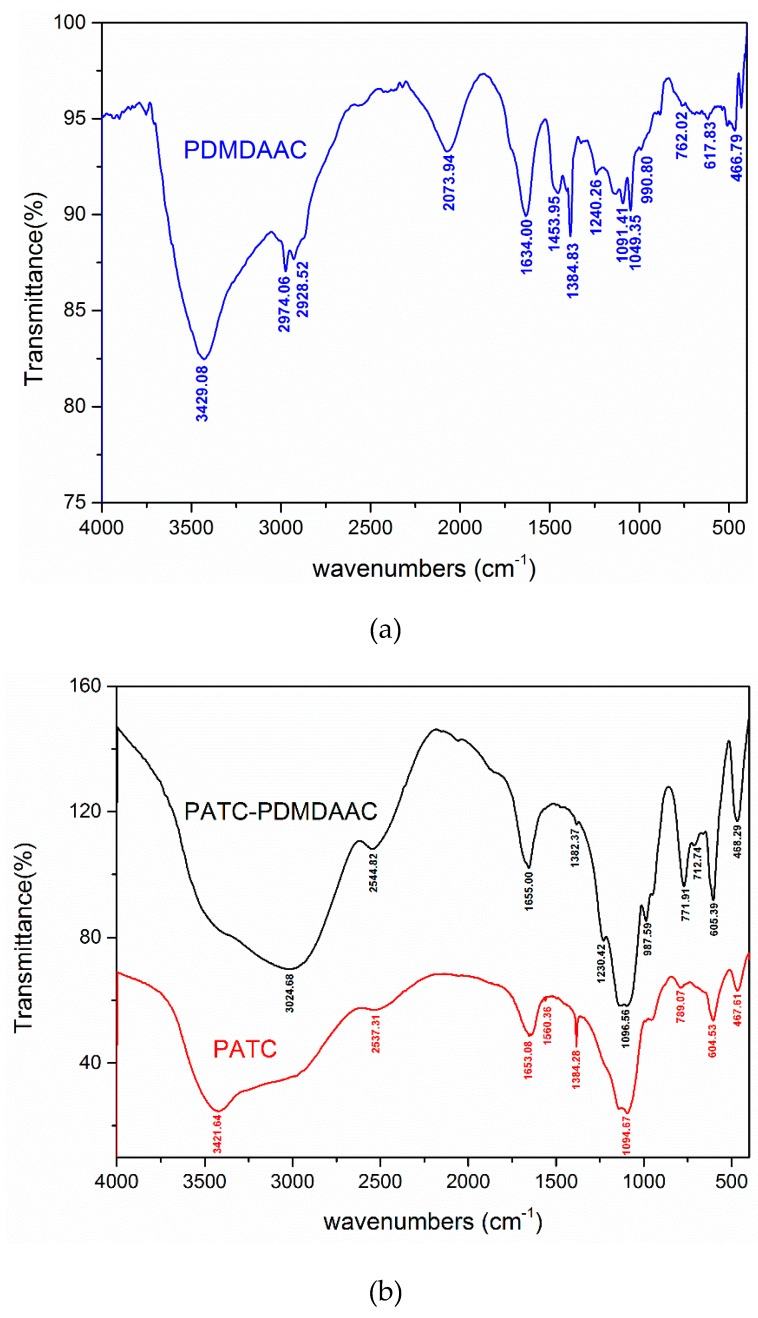
The FT-IR of PATC-PDMDAAC, PATC, and PDMDAAC. (**a**) The FT-IR of PDMDAAC; (**b**) the FT-IR of PATC-PDMDAAC and PDMDAAC.

PDMDAAC and PATC were measured for the analysis of the functional group information of PATC-PDMDAAC and FTIR of PATC-PDMDAAC. The results are shown in Figure 1. The figure shows that the structure of composite flocculant was changed through comparing the infrared images of the three samples. The blue shift of the characteristic adsorption peaks around 3024.68 cm^−1^ in the spectrum of PATC-PDMDAAC was attributed to the vibration absorption peak overlap between N-H and C-H in PDMDAAC. This peak overlap caused the PATC-PDMDAAC to have stretching vibration peaks corresponding to C=C and N-H bonds [14,15]. The peak generated by PATC-PDMDAAC at 1655.00 cm^−1^ was red-shifted compared with the peak generated by PATC at 1653.08 cm^−1^, and the absorption peak of PATC-PDMDAAC was enhanced possibly because of the enhanced bonding of coordination water and central ions in the composite flocculant and the superposition of the absorption peak of the C=O bond [16]. The adsorption peak of 1384.28 cm^−1^ in the PATC (Al-O-Al and Si-O-Si) and the peak at 1384.83 cm^−1^ in PDMDAAC were compounded and transformed into a weak absorption peak in the 1380 cm^−1^ in PATC-PDMDAAC. This phenomenon can be due to the breaking of the Si-O-Si bond in PATC and the combining of Si with methylene in PDMDAAC to form the C-Si bond [17,18]. The absorption peak at 771.91 cm^−1^ was also enhanced. The strong peak at 987.59 cm^−1^ of PATC-PDMDAAC was ascribed to the vibration absorption of –C-H [19]. The peaks at 789.07 cm^−1^ of PATC was sourced from the characteristic groups of the stretching vibration of Ti–O–Ti, which was from the tetravalent complex produced by Ti ion hydrolysis. However, the peak at 771.91 cm^−1^ of the composite flocculant showed a sharp and strong vibration absorption peak and blue shift compared with the PATC. Such an occurrence may be caused by the formation of C-Cl bond between PDMDAAC and PATC. This finding showed that PATC and PDMDAAC are not just simple physical and mechanical mixing, and their bonds interact and transform, resulting in the change of their chemical environment.

#### 3.1.2. XRD Analysis

**Figure 2:**
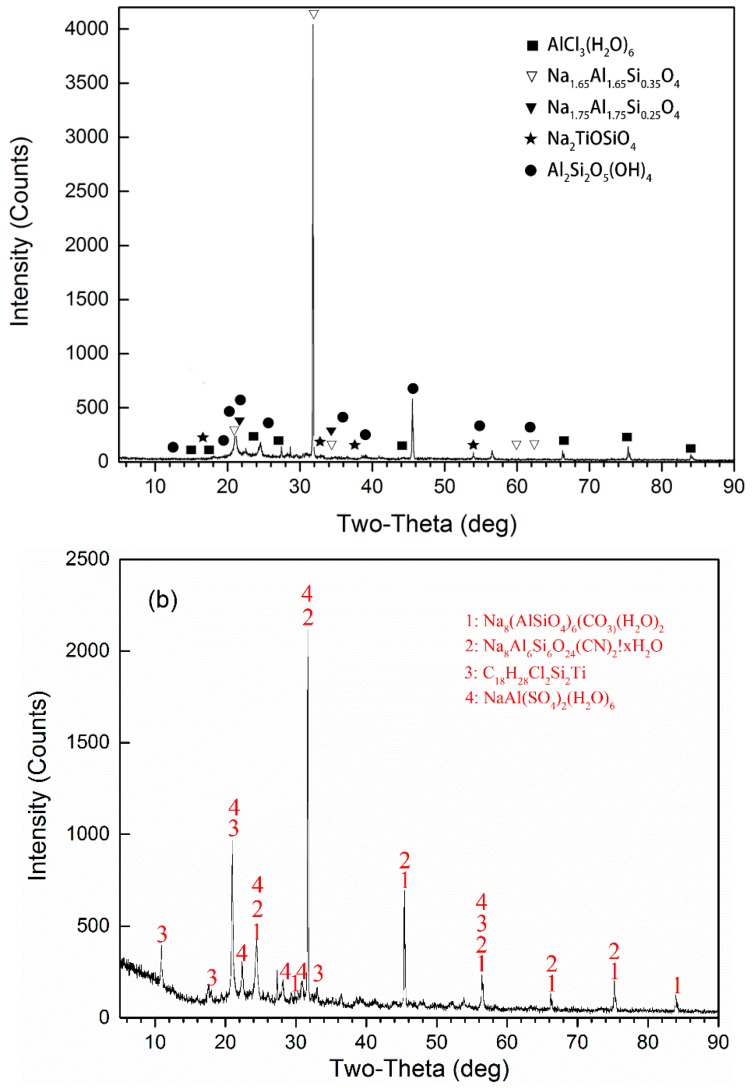
The XRD of PATC (**a**) and PATC-PDMDAAC (**b**).

The XRD spectra of PATC and PATC-PDMDAAC (Figure 2) were used in the investigation of crystal information in PATC-PDMDAAC. The composite flocculant after grinding and drying was scanned with a D8 ADVANCE XRD and then analyzed with Jade 6 software (Jade 6, Materials Data Inc., Livermore, CA, USA), and the possible phase composition was compared with the standard color chart. The profiles of PATC-PDMDAAC showed the peak at 2θ of 11.8° and 18.2°, which did not appear in the XRD diagram of PATC owing to the introduction of PDMDAAC, which formed C_18_H_28_Cl_2_Si_2_Ti with PATC. The composite flocculant XRD pattern was compared and analyzed with the standard color chart. The result showed that the main phases in PATC-PDMDAAC were Na_8_(AlSiO_4_)_6_(CO_3_)(H_2_O)_2_, Na_8_Al_6_Si_6_O_24_(CN)_2_H_2_O, and NaAl(SO_4_)_2_(H_2_O)_6_, which were different from the main phases of PATC. This outcome was attributed to the introduction of PDMDAAC, which changed the composition of each substance and the corresponding chemical reaction that occurred between substances. The result was in agreement with the infrared analysis results, which showed that the two were not simply mechanically mixed.

#### 3.1.3. SEM-EDS Analysis

**Figure 3:**
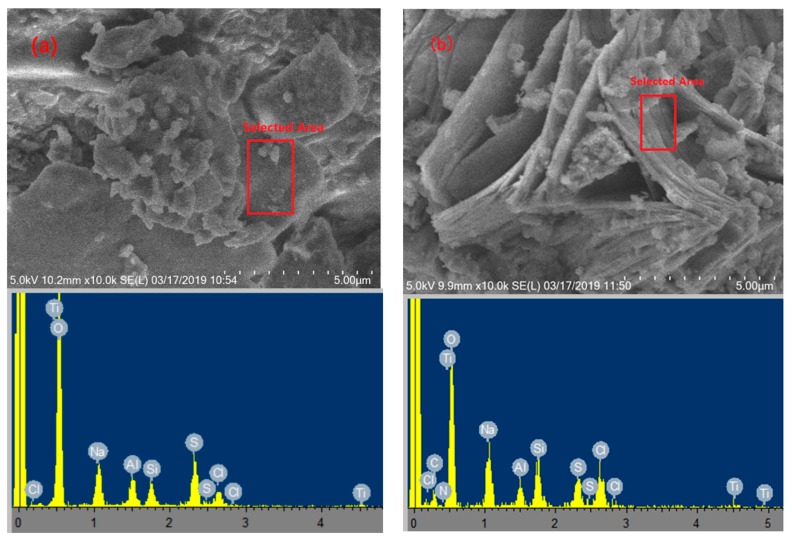
The SEM-EDS of PATC (**a**) and PATC-PDMDAAC (**b**).

SEM is a direct and accurate method for observing the surface structures of materials. In this study, the flocculant (PATC) and (PATC-PDMDAAC) were analyzed by SEM, and the element types and content of the two materials were analyzed by energy dispersive spectrometry. As shown in Figure 3, the inorganic flocculants were mainly distributed in the form of granules, whereas the composite flocculants showed complex structures, including irregular pore and reticular structures. This difference in structure may be mainly attributed to the introduction of PDMDAAC, which enabled the composite flocculant to play an important adsorption bridging role and, thus, further improved the coagulation effect. The energy spectrum showed that the composite flocculant introduced C and N elements compared with the inorganic flocculant PATC because of the addition of C and N elements in PDMDAAC, indicating that a reaction occurred between PATC and PDMDAAC.

#### 3.1.4. TG/DSC Analysis

**Figure 4:**
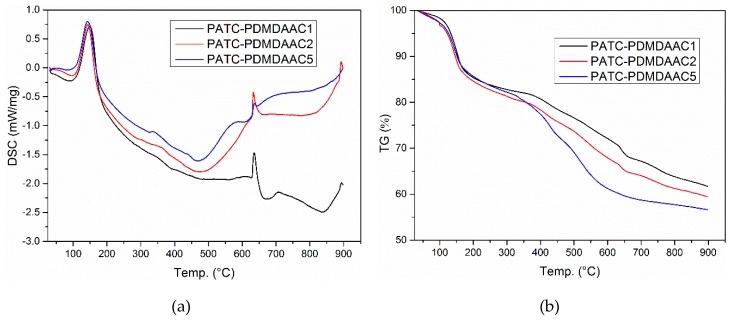
The TG-DSC of PATC-PDMDAAC with various organic/inorganic ratios (organic/inorganic = 0.10 represent PDMDAAC1, organic/inorganic = 0.15 represent PDMDAAC2, organic/inorganic = 0.30 represent PDMDAAC5). (**a**) the DSC of PATC-PDMDAAC; (**b**) the TG of PATC-PDMDAAC.

In this study, the composition and stability of PATC-PDMDAAC were analyzed by thermogravimetric analysis and differential scanning calorimetry. Figure 4 shows the TG and DSC curves of the composite flocculant PATC-PDMDAAC prepared with different inorganic and organic ratios (m (PDMDAAC)/m (PATC) = 0.1 for PATC-PDMDAAC1, m (PDMDAAC)/m (PATC) = 0.15 for PATC-PDMDAAC2, and m(PDMDAAC)/m(PATC) = 0.3 for PATC-PDMDAAC5). The thermal decomposition of the samples showed three well-defined steps. In the first stage, the weight loss of PATC-PDMDAAC1, PATC-PDMDAAC2, and PATC-PDMDAAC5 were observed at the ranges of 30.00–175.28 °C, 30.00–166.33 °C, and 30.00–164.32 °C, and the ratios of weight loss were 13.169%, 13.327%, and 12.596%, respectively. The corresponding absorption peaks were 88.58 °C, 97.38 °C, and 81.18 °C. The weight loss in this period was considered to have been caused by the evaporation of crystallized water in the flocculant [20]. The second stage of weight loss rate for flocculants showed 16.288%, 20.019%, and 18.649%, and its corresponding absorption peak was at approximately 470 °C; the main reason for the weight loss in this stage may be that the chemical bonds in the samples were broken and the structure of the flocculant began to change [21]. In the third stage, the weight loss were 8.918% for PATC-PDMDAAC1, 7.280% for PATC-PDMDAAC2, and 12.223% for PATC-PDMDAAC3. The main reason for the weightlessness of this section may be that the main chain of the sample begins to undergo thermal decomposition, and the molecular chain breaks down, leading to the reduction of mass [20]. The final residual masses of the three samples were 61.625%, 59.374%, and 56.532%, respectively. The residual mass of their final products was more than 50%, indicating that the samples showed good stability.

#### 3.1.5. Morphological Analysis of Aluminum

**Figure 5:**
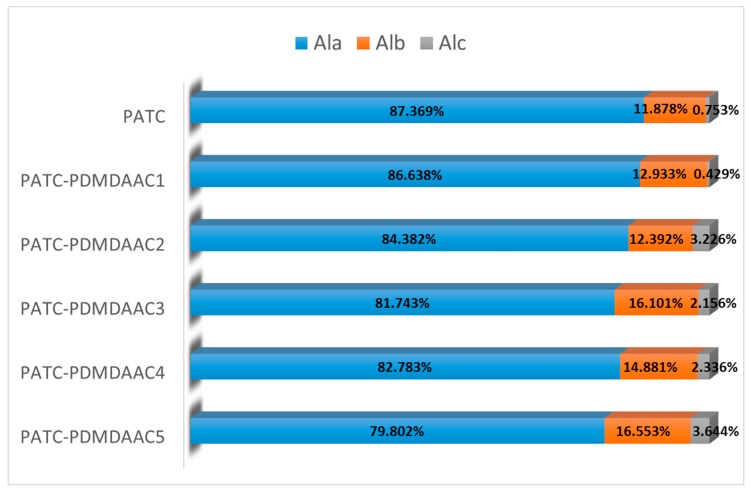
Al morphology of PATC-PDMDAAC with different material ratios (organic/inorganic = 0.10 represent PDMDAAC1, organic/inorganic = 0.15 represent PDMDAAC2, organic/inorganic = 0.20 represent PDMDAAC3, organic/inorganic = 0.25 represent PDMDAAC4, organic/inorganic = 0.30 represent PDMDAAC5).

The morphological distribution of Al in the composite flocculant directly affects flocculant performance, whereas that of aluminum may be related to organic components, alkalization degree, proportion of organic and inorganic components, and other factors [22]. The Al-ferron-timed complex colorimetric method is a common and effective technique to study the morphological distribution of Al in hydrolyzed aluminum solution. The morphology of Al was divided into three categories on the basis of the difference in complexation reaction between the colorant ferron and various morphologies after the hydrolysis of aluminum. Ala (monomeric Al species) reacted with ferron in 1 min, Alb (polymeric Al species) reacted between 1 and 120 min, and Alc (colloidal Al species) reacted with ferron within 24 h [23]. In this study, the PATC-PDMDAAC composites with different material ratios were morphologically analyzed. The results are shown in Figure 5. The figure illustrates that the Al in PATC-PDMDAAC mainly exists in the form of Ala. The Ala content of composite flocculant decreased from 87.369 to 79.802% with the increase of PDMDAAC. However, the content of polynuclear hydroxyl complex Alb slightly increased. The Alc content in the form of aluminum polymer also increased compared with PATC. The influence of PDMDAAC in coagulants on the Al morphological distribution is determined by the high pH of PDMDAAC. The addition of PDMDAAC changed the system pH. Increasing pH to a certain extent will promote the reaction between the medium polymer (Alb) and polymer macromolecule (Alc). The PDMDAAC-induced change in Al morphology further indicated that PATC and PDMDAAC are not simply physical mixtures.

### 3.2. Flocculation Properties

#### 3.2.1. Effect of dosage

**Figure 6:**
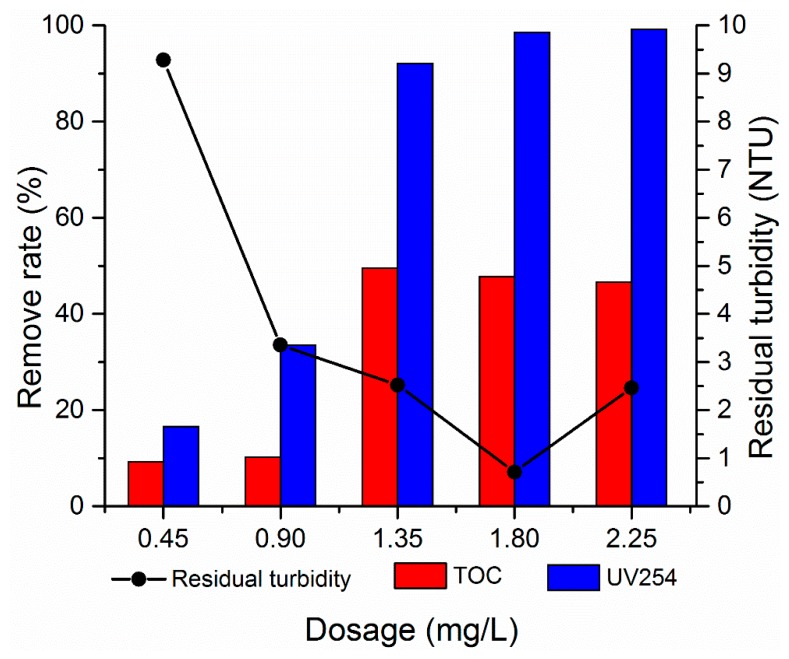
Effect of PATC-PDMDAAC dosage on flocculation.

Flocculation is a physicochemical reaction in which a series of equilibrium reactions occurs. The dosage of flocculant has a great influence on the water treatment effect. The effect of PATC-PDMDAAC dosage on flocculation was investigated to evaluate the flocculation performance of PATC-PDMDAAC by controlling a single variable. The PATC-PDMDAAC dosage of 0.45–2.25 mg/L was added to the simulated water. The treatment effect trend after coagulation and precipitation is shown in Figure 6. The turbidity removal efficiency rapidly increased with the increase of PATC-PDMDAAC dosage before 1.80 mg/L. The turbidity decreased to 0.71 NTU at the dosage of 1.80 mg/L and slowly increased with further dosage increase. The 1.35 mg/L dosage had a great treatment effect on TOC of low turbidity and temperature water, and its removal rate was 49.57%. The optimal dosage of UV254 treatment was 2.25 mg/L, and the removal rate was 99.23%. This phenomenon may be due to the Al and Ti ions in the water that were hydrolyzed after PATC-PDMDAAC was added into the water, and the polymerized hydroxyl complex formed in the water collided and neutralized with the negatively-charged particles in the water, thus making the colloid unstable. In addition, PDMDAAC could also be extended to enhance the adsorption and bridging of suspended substances in the water, resulting in an enhanced treatment effect [18,24]. With the further dosage increase, the treatment effect of the coagulant on low-temperature and low-turbidity water was reduced, which may be because a large number of positively-charged hydroxyl complexes were ionized in the water with the further dosage increase. This condition resulted in the re-stabilization of colloidal particles after adsorption and stabilization, resulting in the turbidity increase [25]. When the dosage was 1.80 mg/L, the treatment effects on TOC and UV254 were slightly reduced compared with the optimal treatment effect alone, but it had a good treatment effect on turbidity. Therefore, the dosage of 1.80 mg/L was selected as the optimal treatment effect.

#### 3.2.2. Effect of Different m(PDMDAAC)/m(PATC)

**Figure 7:**
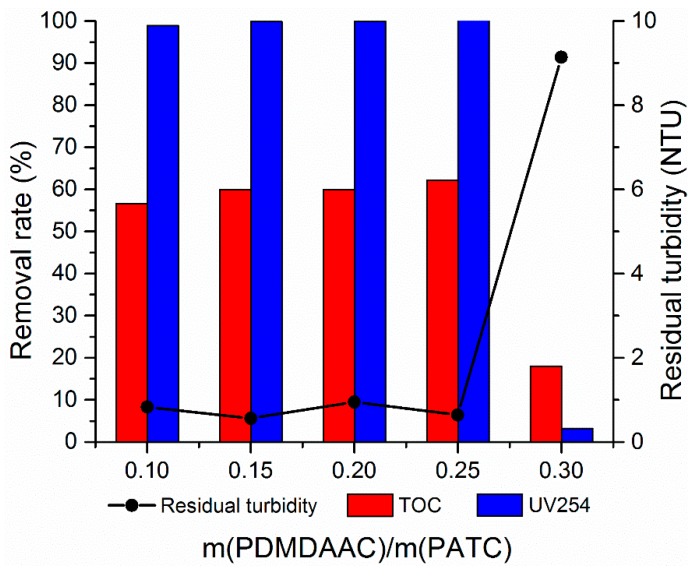
Effect of different m(PDMDAAC)/m(PATC) on flocculation.

For inorganic–organic compound flocculants, the reasonable ratio of organic and inorganic flocculants is the guarantee of obtaining a good flocculation effect. The dosage of PATC-PDMDAAC was fixed at 1.80 mg/L. Composite flocculants prepared with different m(PDMDAAC)/m(PATC) were used to treat the simulated low-temperature and low-turbidity water. The treatment effect is shown in Figure 7. The results showed that the composite flocculant had a better and stable effect on low-temperature and low-turbidity water when the ratio of organic and inorganic was 0.1–0.25. PATC-PDMDAAC with a composite ratio of 0.15 had the optimal treatment effect on low-temperature and low-turbidity water. The turbidity removal rate could be reduced to 0.56 NTU. The removal rates of TOC and UV254 reached 59.89% and 99.88%, respectively. When the composite ratio was 0.25, the treatment effect on TOC and UV254 slightly increased, but the treatment effect on turbidity decreased. This occurrence is due to the cations with quaternary ammonium salts in the five-membered ring structure of PDMDAAC that show high density positivity and react with PATC after they are compounded. The ionic Ti and Al in PATC transformed to polymerization morphology, and the –CH_3_ existing around the pyrrole ring structure of PDMDAAC showed a barrier protection effect on positive charge. Accordingly, the potential of the solution to shrink the dual layer increased, and the long chain structure of PDMDAAC produced an adsorption bridging effect, thereby further improving the treatment effect of low-temperature and low-turbidity water [26,27]. Increasing the PDMDAAC content in a certain range was beneficial to the electric neutralization. However, the results showed that the treatment effect of the composite flocculant on low-temperature and low-turbidity water showed a sharp decrease when the ratio of organic and inorganic was 0.30. The residual turbidity was 9.14 NTU, and TOC and UV254 removal rates were reduced to 17.98% and 5.43%, respectively. This finding may be attributed to the organic part of the coagulant that exhibited a particularly strong sweep capacity due to the high organic content. The electroneutralization effect of suspended particles in low-temperature and low-turbidity water with inorganic part in coagulant and the adsorption bridging part with organic part did not reached the balance. However, the curling-sweep effect with organic part begins to occur, thereby leading to the insufficient performance of electroneutralization and adsorption bridging ability; consequently, the treatment effect of PATC-PDMDAAC on low-temperature and low-turbidity water was affected [28]. In combination with economic considerations, the ratio of organic and inorganic of 0.15 to prepare PATC-PDMDAAC was selected as the optimal preparation condition. Therefore, the ratio of organic to inorganic compounds in a certain range should be improved.

#### 3.2.3. Effect of pH

**Figure 8:**
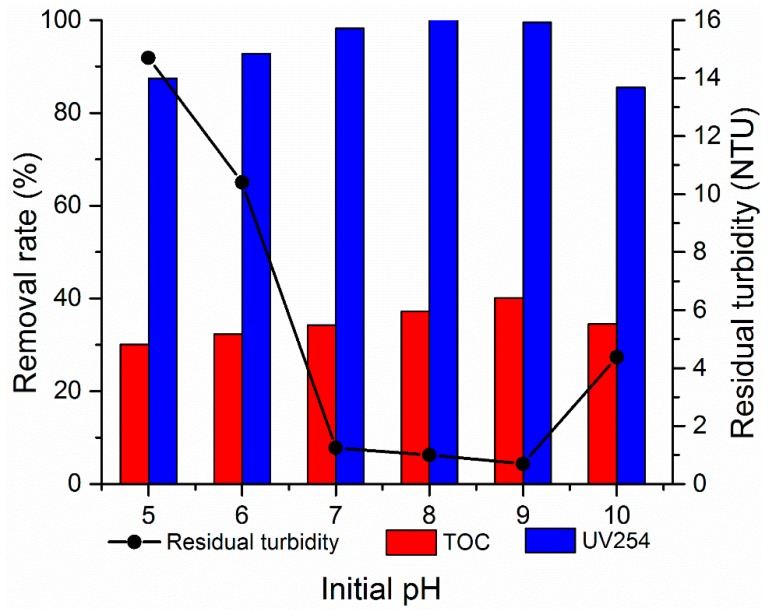
Effect of initial pH of water sample on the flocculation effect.

The pH of raw water is one of the important factors affecting the flocculation effect; the pH affects flocculation by influencing the potential of colloidal particles in water [29]. In this section, the effects of different pH values on the removal efficiency of turbidity, TOC, and UV254 were investigated. The 0.01 mol/L NaOH and 0.01 mol/L HCl solutions were adopted to adjust the pH of simulated wastewater. Approximately, 1.80 mg/L composite flocculant PATC-PDMDAAC (the ratio of organic and inorganic was 0.15) was added to the simulated water with different pH values. The results are presented in Figure 8. The figure shows that the removal rates of turbidity, TOC, and UV254 first increased and then decreased with the pH increase. Turbidity increased after coagulation and precipitation under the condition of solution pH < 7.0. The removal rate of UV254 was lower than 90%. This phenomenon was attributed to the inorganic components of the composite flocculant PATC-PDMDAAC that mainly exists in the form of hydrated ions in the solution when the pH value is low, and their adsorption capacity is weak. Several colloidal particles in the suspension were adsorbed, and a large number of particles remained in the upper liquid, resulting in the floc formation with a small particle size and the supernatant with high turbidity [30]. With the increase of pH value, the treatment effects of PATC-PDMDAAC on turbidity, UV254, and TOC accordingly increased. When the pH value was nine, the removal rates of turbidity, TOC, and UV254 were 93% (0.7 NTU), 40.16%, and 99.58%, respectively. Several reasons may explain these results. By contrast, the amount of –OH in the water increased with the pH increase. The hydration ions further hydrolyze to form polynuclear hydroxyl complex ions with high degree of polymerization. The mononuclear hydroxyl group of Al(III) was further polymerized through collision to form polynuclear hydroxyl complex Al_x_(OH)_y_^(3x−y)+^, which plays an important role in electroneutralization. On the contrary, increasing the reaction pH can contribute to the degree of polymerization of polysilicic acid, Al^3+^, and Ti^4+^, neutralize the charge, reduce the electrostatic repulsion, and shorten the distance between the particles. Accordingly, the bridging connection of the long polymer chain to the particles was facilitated. The –C=O and –NH_2_ in the polymer long chain formed hydrogen bond adsorption with –OH or –O on the surface, and the particles were adsorbed through the bridging connection between particles [31]. However, the removal efficiency of turbidity, UV254, and TOC sharply decreased when the pH value was greater than nine. This occurrence may be due to polynuclear hydroxyl complex ions, which formed water-insoluble Al(OH)_3_ under strong alkali when the pH value was particularly high. Consequently, the adsorption can only be partially exerted, and the effect of treatment was reduced. The comprehensive analysis indicated that the optimal pH reaction condition was selected to treat low-temperature and low-turbidity water as a coagulant when the pH of the simulated water sample was 9.0.

#### 3.2.4. Effect of Sedimentation Time

**Figure 9:**
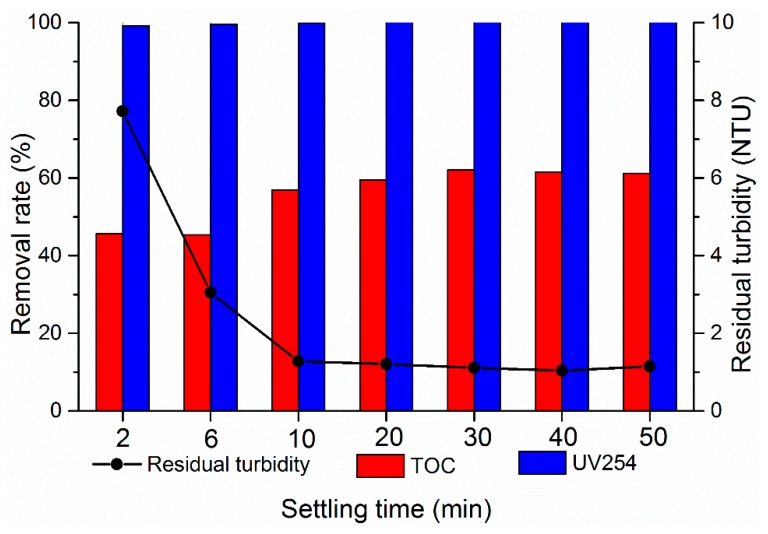
Effect of sedimentation time on flocculation.

This study further investigated the effect of precipitation time on low-temperature and low-turbidity water treatment to determine the influence of sedimentation time on flocculation effect. The 1.8 mg/L dosage of PATC-PDMDAAC was added into the 9.0 pH of simulated water. Seven different precipitation times (2, 6, 10, 20, 30, 40, and 50 min) were selected. As shown in Figure 9, the removal effect of coagulants was also improved with the extension of precipitation time. The treatment effects of turbidity and TOC were unsatisfactory when the precipitation time was less than 10 min. This condition was because of the short precipitation time and floc formation after coagulation did not completely accumulate into a group and sink, leading to poor flocculation effect. The coagulant and part of the flocs were partially extended, and the adsorbed suspended particles were far from reaching the saturated state. This condition is one of the reasons for the poor treatment effect caused by short precipitation time. The removal effects of turbidity, TOC, and UV254 were basically stable when the precipitation time was more than 30 min. At this time, the flocculant adsorption was basically completed. When the precipitation time was 40 min, the turbidity was reduced to the lowest value of 1.04 NTU. The removal rates of UV254 and TOC were up to 99.99% and 62.06%, respectively.

#### 3.2.5. Effect of Stirring Speed

**Figure 10:**
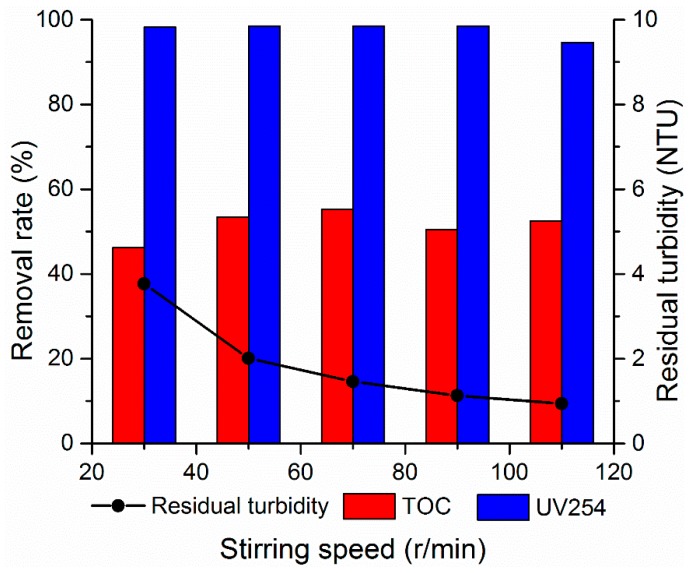
Effect of stirring speed on flocculation.

While the stirring speed is particularly fast during flocculation, then it will break the large solid particles into small ones. This occurrence will cause particles that can be precipitated to be crushed into particles that cannot be precipitated [32]. By contrast, if the stirring speed is particularly slow, then the flocculant can only partially contact with solid particles, which is non-conducive to the collection of colloidal particles by the flocculant. The concentration distribution of the flocculant is non-uniform, which is non-conducive to play the role of flocculant. The appropriate mixing speed is conducive to improve the flocculation effect. Therefore, the effect of stirring speed is investigated in this section. The initial pH of the simulated water sample is fixed at 9.0, and the coagulant with an organic and inorganic ratio of 0.15 is added at 1.8 mg/L. In the same rapid mixing process, the influence of the stirring speed values of 30, 50, 70, 90, and 110 r/min on the flocculation effect is investigated, and the results are presented in Figure 10. As shown in Figure 10, the rapid stirring has an enhanced effect in terms of turbidity removal effect. At the stirring speed of 110 r/min, the turbidity dropped to the lowest value of 0.94 NTU. The treatment effect of UV254 is relatively stable, and the removal effect reached more than 90%. Rapid stirring can accelerate the contact between the coagulant and the colloidal particles in water. When –COOH in humic acid molecules complexates or chelates with Ti^4+^ and Al^3+^ or hydrolysates, the double-layer of colloidal particles is compressed to provide full influence to the effect of electroneutralization [33]. In the stirring process, the long chain of PDMDAAC can be adsorbed and linked by electrostatic attraction, van der Waals force, and hydrogen bond force through the active parts and the colloidal particles. The fine suspended substances are incompletely destabilized; thus, the flocs can realize further collision and aggregation and form larger flocs [34]. The removal effect of TOC first increased and then decreased. The optimal treatment effect is achieved at a stirring speed of 70 r/min, and the removal rate is 55.19%. If the mixing speed is particularly fast, then the aggregates will be broken. The long chain of the polymer will be also broken to reduce the coagulation effect. In combination with the treatment trend of turbidity and economic benefits, the stirring rate is controlled at 110 r/min.

### 3.3. ZP Analysis

**Figure 11:**
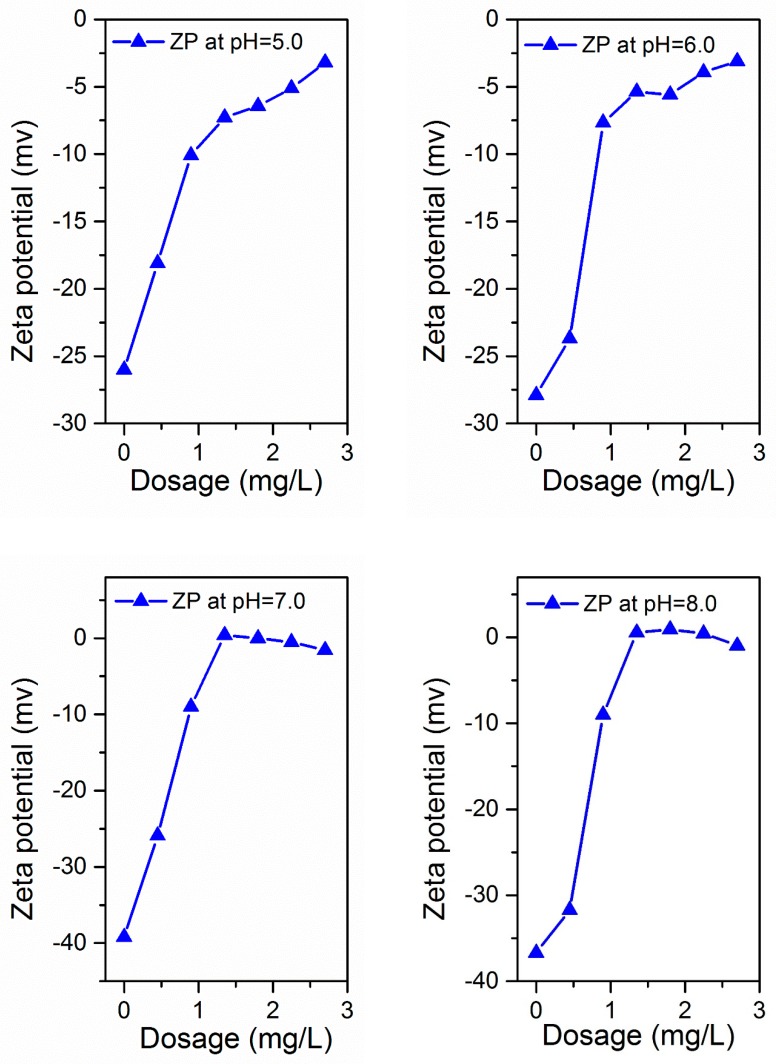

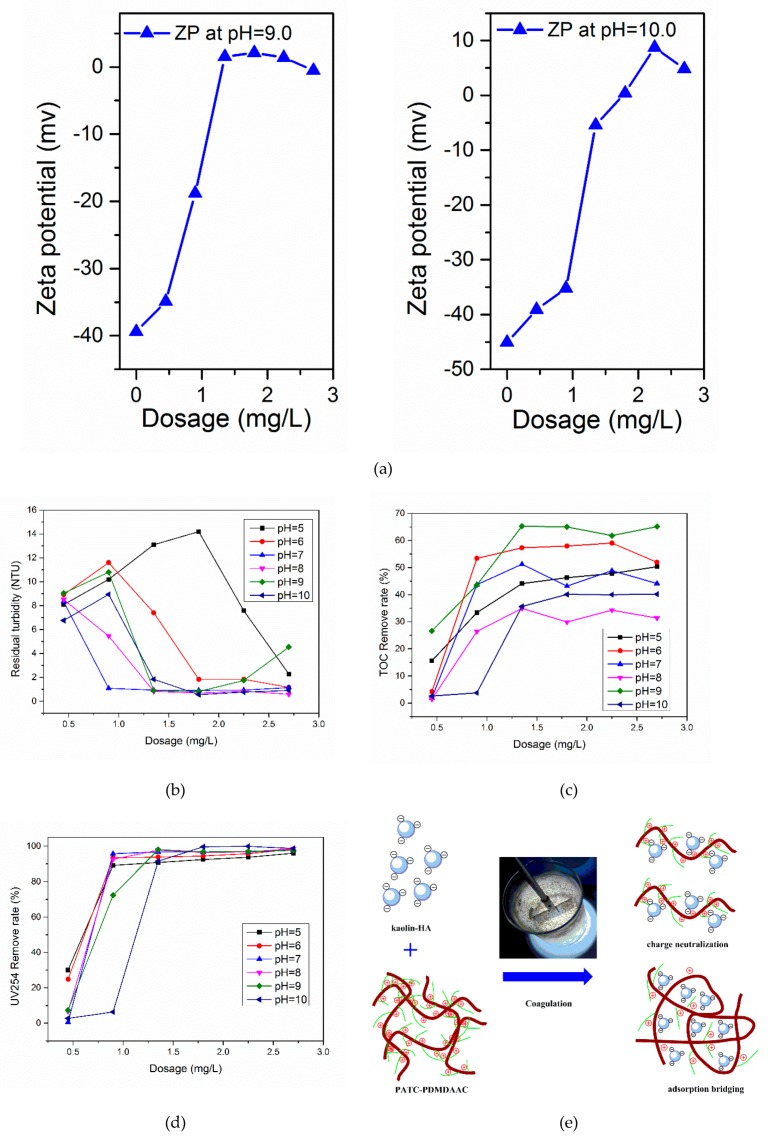
Zeta potential analysis diagram and treatment effect under different pH and dosage: (**a**) zeta potential analysis, (**b**) turbidity treatment effect, (**c**) TOC treatment effect, (**d**) UV254 treatment effect, and (**e**) mechanism diagram of the coagulation process).

The charge condition of the flocculant and its reaction process is also a key factor to explain the electroneutralization performance of the flocculant. The reaction mechanism is usually understood in the ZP form. The ZP of supernatant after flocculation was investigated with different dosages of PATC-PDMDAAC at various pH environments to effectively understand the flocculation process and mechanism of PATC-PDMDAAC. The removal effects of turbidity, UV254, TOC, and ZP were analyzed to investigate the reaction mechanism. The results are displayed in Figure 11a–d, and the ZP of the prepared composite flocculant was approximately 3.75 mv, which is higher than that of the inorganic flocculant [18]. This condition was attributed to PDMDAAC, which is a cationic organic polymer composite flocculant, with a positive charge on its surface. Therefore, the potential will increase when combined with PATC. As illustrated in Figure 11a, the ZP value of simulated water varied from −26 mv to −45.1 mv before flocculant addition with the pH increase. The ZP increased with flocculant dosage under acidic environments. Such ZP increased from −26 mv to −3.2 mv with the increase in PATC-PDMDAAC dosage from 0 to 2.7 mg/L at pH 5.0. The ZP first increased and then decreased with the increase in flocculant dosage under neutral and alkaline environment, and it changed from negative to positive as the dosage increased. The maximum ZP (8.77 mv) was observed at pH of 10.0 and dosage of 2.25 mg/L. The ZP increase was due to electroneutralization between the added PATC-PDMDAAC and the negatively charged humic acid-kaolin particles [35]. Figure 11b–d shows the effects of turbidity, TOC, and UV254 under different pH and dosage conditions. The figure shows that the treatment effect of turbidity and TOC was worse in an acidic environment than that in neutral and alkaline conditions. The pH for the treatment effect of UV254 exhibited little effect on the removal rate after the dosage was greater than 1.35 mg/L. Comprehensive analysis of the treatment effect of coagulant on low-temperature and low-turbidity water showed that the treatment effect was optimal when the pH value was nine, and the dosage was 1.80 mg/L. The removal rates of TOC and UV254 were 96.68% and 65%, respectively, and the turbidity can be reduced to 0.81 NTU. The corresponding ZP was 2.1 mv. The ZP was greater than zero when the treatment effect reached its maximum. This finding indicated that PATC-PDMDAAC has not only an electric neutralization effect but also an adsorption effect in the treatment of low-temperature and low-turbidity water. This result was consistent with the previous analysis that Al accounts for a large proportion in morphological analysis of aluminum coagulant and can play a better role in electrical neutralization. Combined with the treatment effect and ZP result analysis, PATC-PDMDAAC treatment of low-temperature and low-turbidity water may be the main role of electrical neutralization and adsorption bridge.

### 3.4. 3D Fluorescence Spectroscopy Analysis

**Figure 12:**
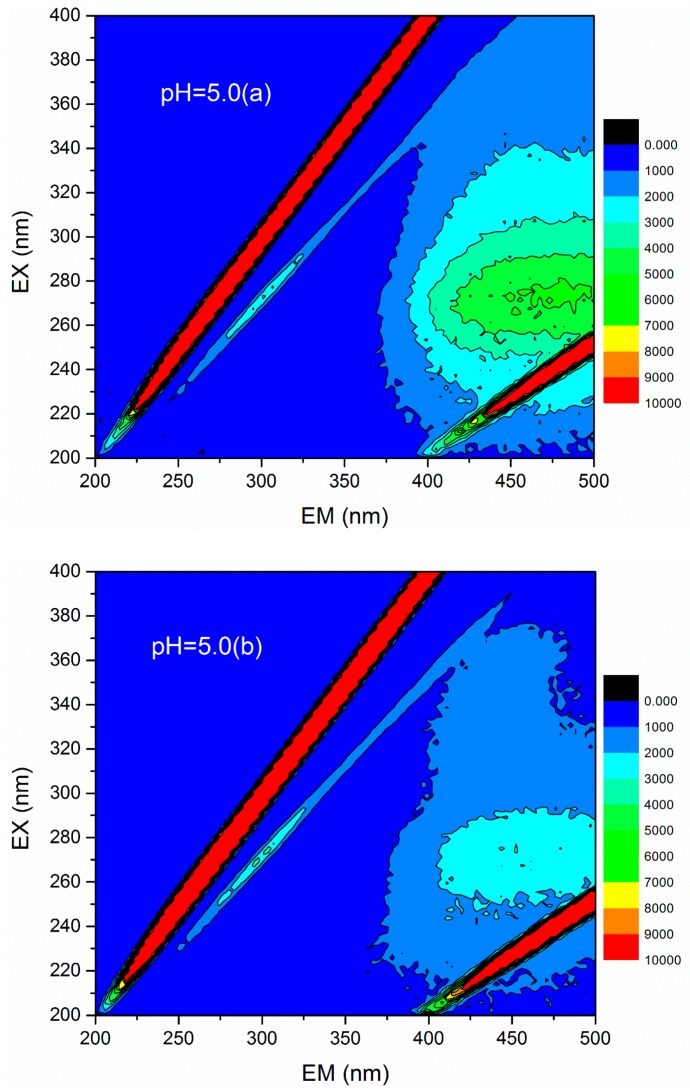

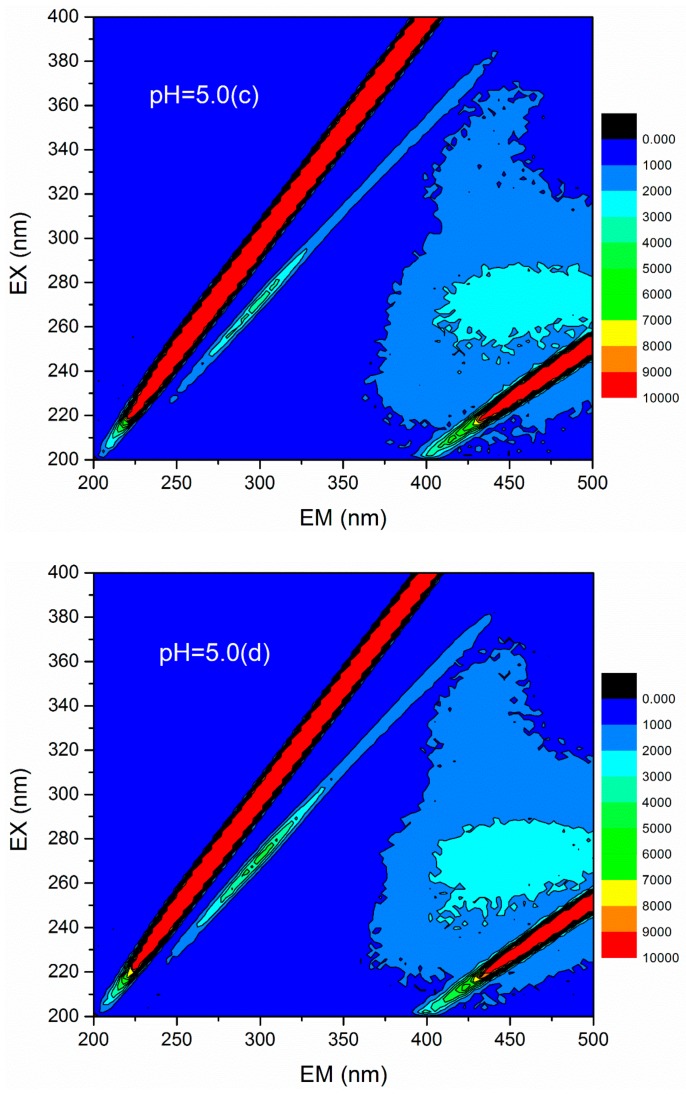

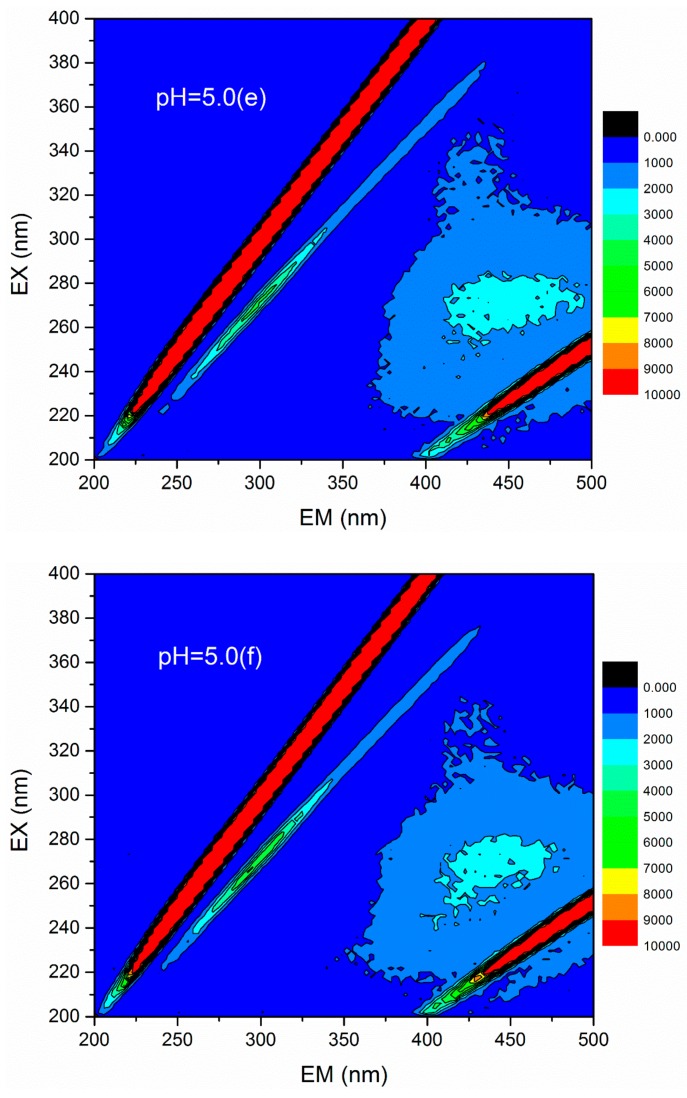

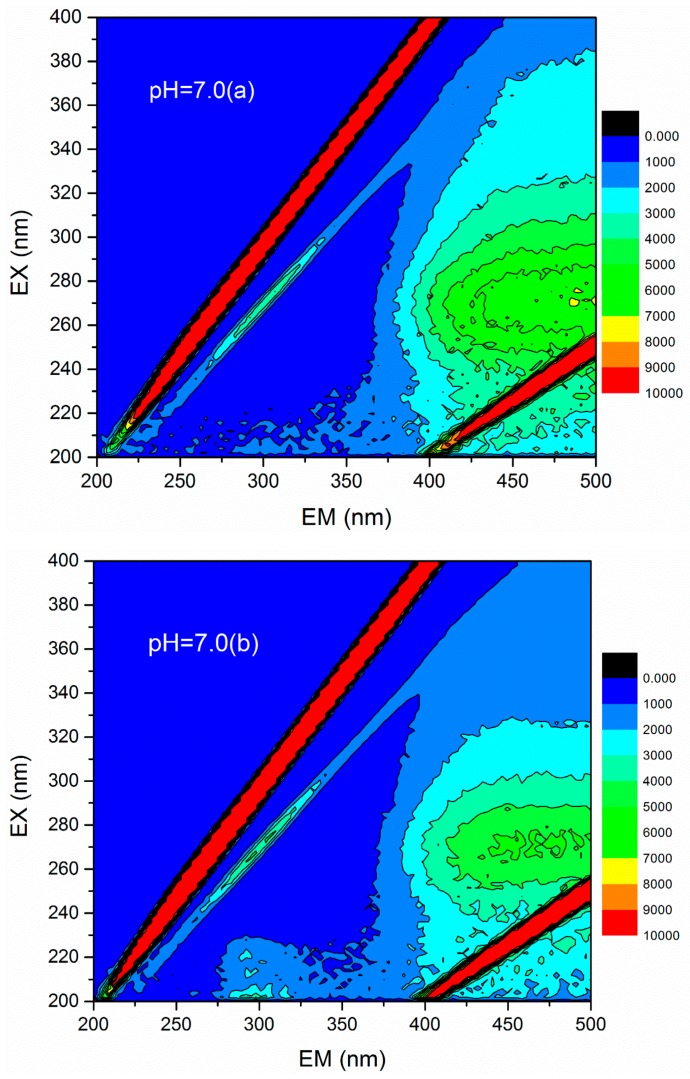

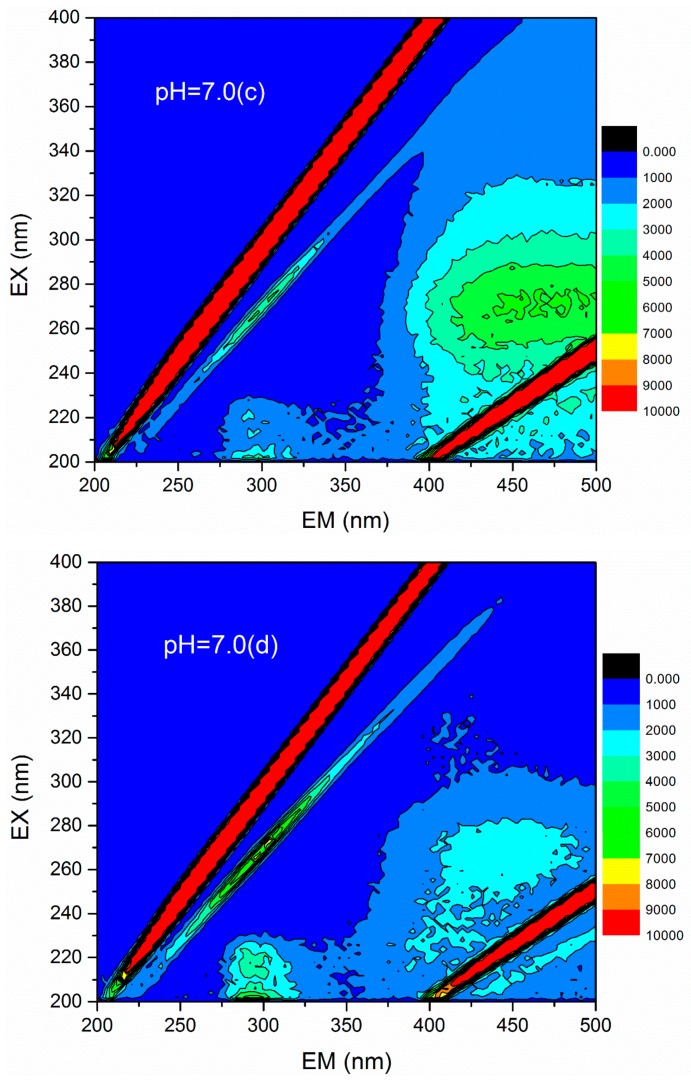

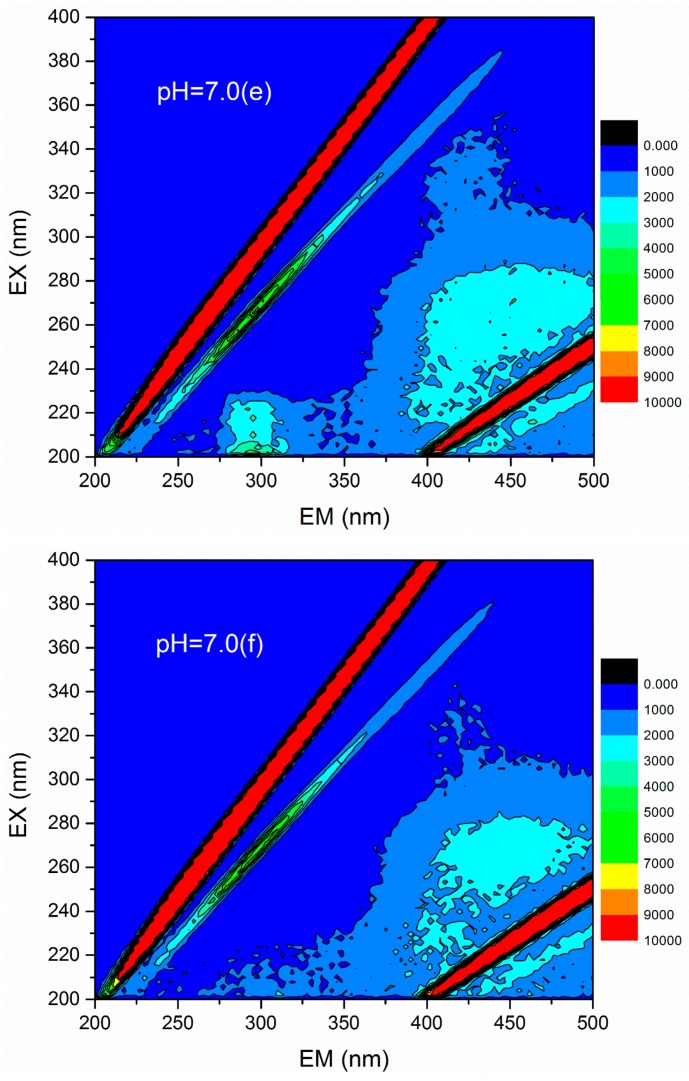

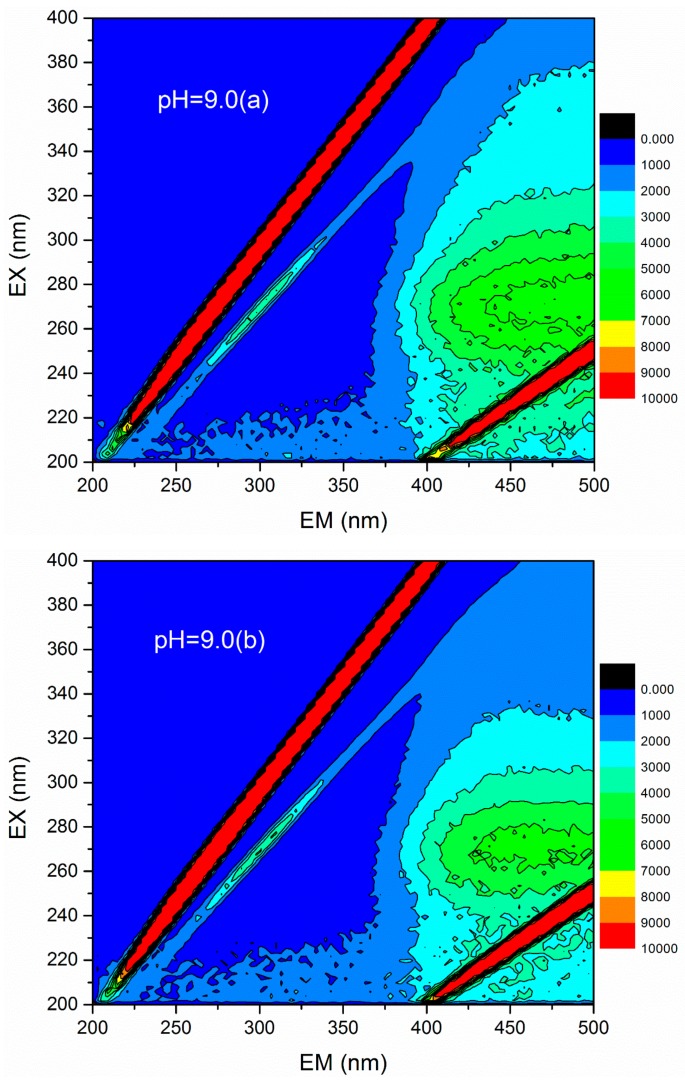

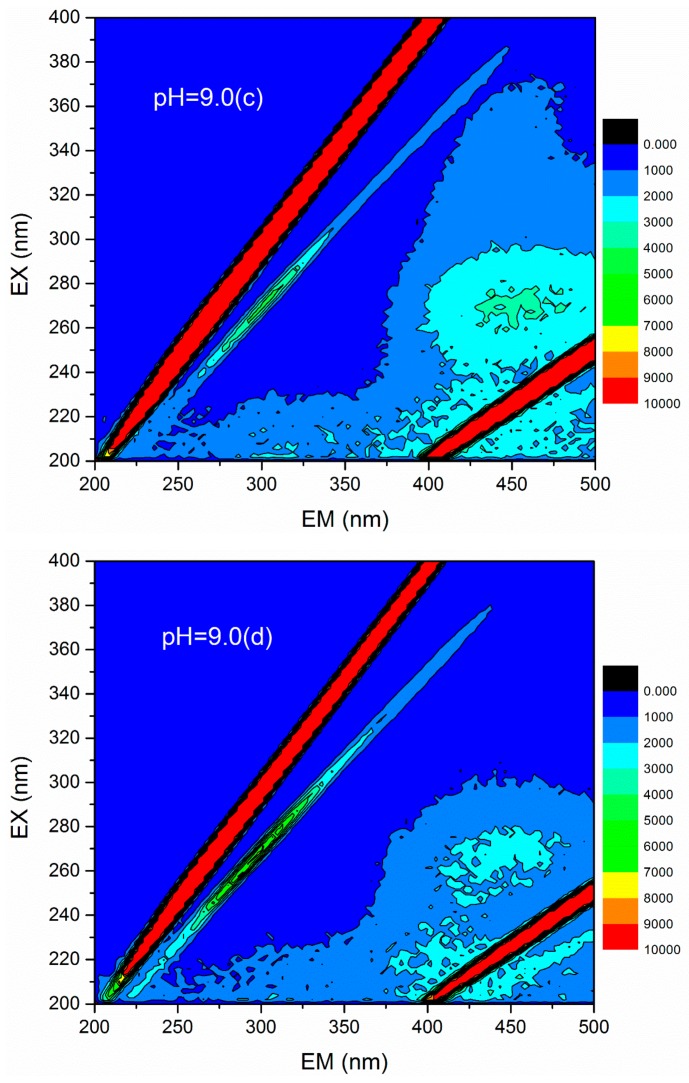

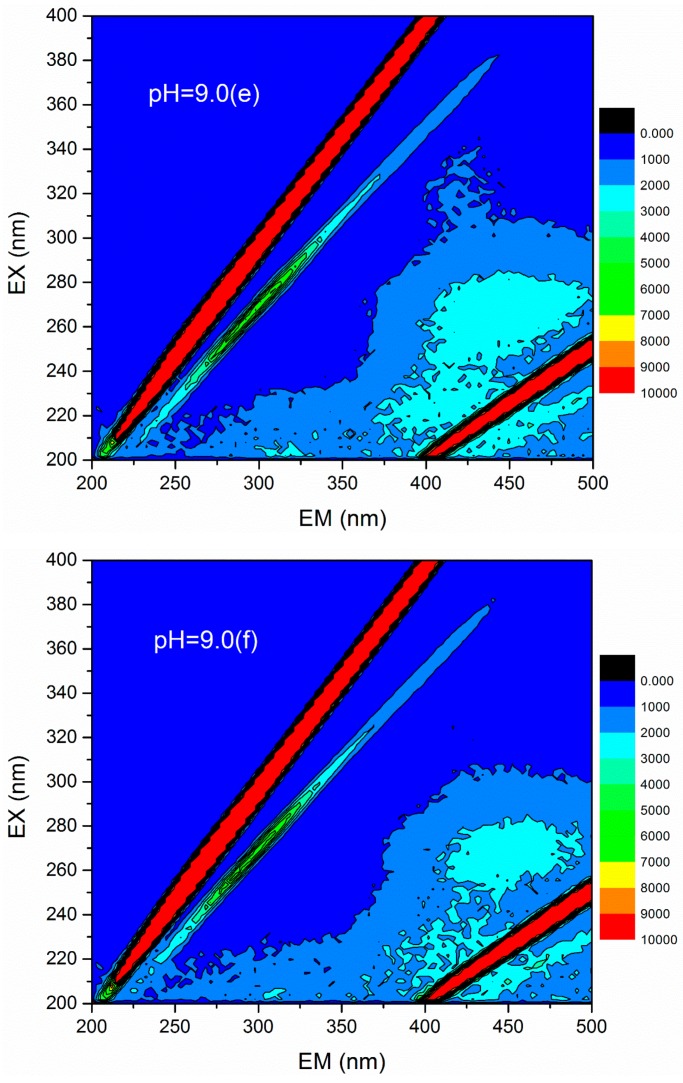
Three-dimensional fluorescence spectra of raw water with different pH values and different dosages. pH = 5, (**a**)–(**f**) the changes of 3D fluorescence images in simulated water with different coagulant dosages (a = 0 mg/L, b = 0.45 mg/L, c = 0.90 mg/L, d =1.35 mg/L, e = 1.80 mg/L, and f = 2.25 mg/L); pH = 7, (**a**)–(**f**) the changes of 3D fluorescence images in simulated water with different coagulant dosages (a = 0 mg/L, b = 0.45 mg/L, c = 0.90 mg/L, d =1.35 mg/L, e = 1.80 mg/L, and f = 2.25 mg/L); pH = 9, (**a**)–(**f**) the changes of 3D fluorescence images in simulated water with different coagulant dosages (a = 0 mg/L, b = 0.45 mg/L, c = 0.90 mg/L, d =1.35 mg/L, e = 1.80 mg/L, and f = 2.25 mg/L).

3D fluorescence spectroscopy is widely used to quantitatively or qualitatively describe the physical and chemical properties of organic compounds in water due to its various advantages, such as fast test speed, good selectivity, and no damage to the sample structure [36]. The 3D fluorescence spectrum was employed to analyze the HA variations in flocculation of humic acid-kaolin composite water. This approach was initiated to further evaluate the flocculation process. The 3D fluorescence spectra were measured with an F-4600 fluorescence spectrophotometer (Hitachi, Tokyo, Japan). The range of excitation wavelength (EX) was 200–400 nm, the step length was set to 2 nm, and the emission wavelength (EM) ranged from 200 to 500 at 3 nm sampling increments. The scanning speed was controlled as 12,000 nm/min. In this study, the changes of 3D fluorescence images in simulated water with different coagulant dosages (a = 0 mg/L, b = 0.45 mg/L, c = 0.90 mg/L, d =1.35 mg/L, e = 1.80 mg/L, and f = 2.25 mg/L) were investigated under acid, base, and neutral conditions (pH = 5.0, 7.0, and 9.0). The results are shown in Figure 12. The data matrix of 3D fluorescence spectra of simulated water samples was analyzed under different pH conditions and dosages. Accordingly, the corresponding emission/excitation wavelength and fluorescence intensity of the fluorescence peaks after each dosages were obtained, as shown in Table 2. Figure 12 shows that the response value of the 3D fluorescence image of raw water (the dosage of PATC-PDMDAAC was 0 mg/L) was mainly distributed in the EM range = 380–500 nm, and the EX = 250–400 nm, which was mainly the distribution area of a type of humic acid. The fluorescence intensities of 7219 and 7053 at pH values 7.0 and 9.0 were higher than that of 5558 at pH 5.0; this outcome may be due to the dissociation of a large number of acidic functional groups in the humic acid molecules as pH increased, resulting in the bonding electrons being exposed and fluorescence enhancement [37].

With the increase of PATC-PDMDAAC dosage, the fluorescence intensity of the solution gradually decreased. The fluorescence range was reduced to a certain extent. The fluorescence peak position was also shifted to a certain degree. The energy conversion in the reaction process between coagulant and humic acid caused the shift of fluorescence peak position. This finding indicates that PATC-PDMDAAC exhibited a certain degradation effect on humic acid substances. This condition may be caused by the electric neutralization reaction between the negative charge in humic organic matter and the coagulant. When the pH of the original simulated wastewater was 5, and the dosage of PATC-PDMDAAC was 2.25 mg/L, and the removal rate of fluorescence intensity of the solution was up to 53.418%. When the pH values of raw water were 7.0 and 9.0, the dosage of PATC-PDMDAAC was 1.35 mg/L, the fluorescence intensity drops to the lowest value, and the removal rates of fluorescence intensity were 61.186% and 64.100%, respectively. These values were consistent with the optimal removal rate of TOC under the same initial conditions. The above-mentioned analysis indicated that PATC-PDMDAAC had a good treatment effect on humic acid and was in agreement with the TOC treatment effect under the corresponding conditions. This finding indicated that the TOC size showed a good correlation with the fluorescence intensity value. The results showed that the removal effect of fluorescence intensity was improved with the pH increase. This condition was because of the low-charge positive multicore complex ions or metal compound condensates generated by the hydrolysis of the added coagulant that had a bonding and bridging effect on the destabilized particles with the pH increase. Accordingly, the colloidal particles aggregated and precipitated, so the coagulation effect was enhanced. Under the low pH condition, the metal ion hydrolysis in the coagulant was incomplete. The flocs were difficult to form, and the electric neutralization ability could not be given full play. Only the adsorption bridging function of coagulant itself can be relied on.

### 3.5. Kinetic Investigation

**Figure 13:**
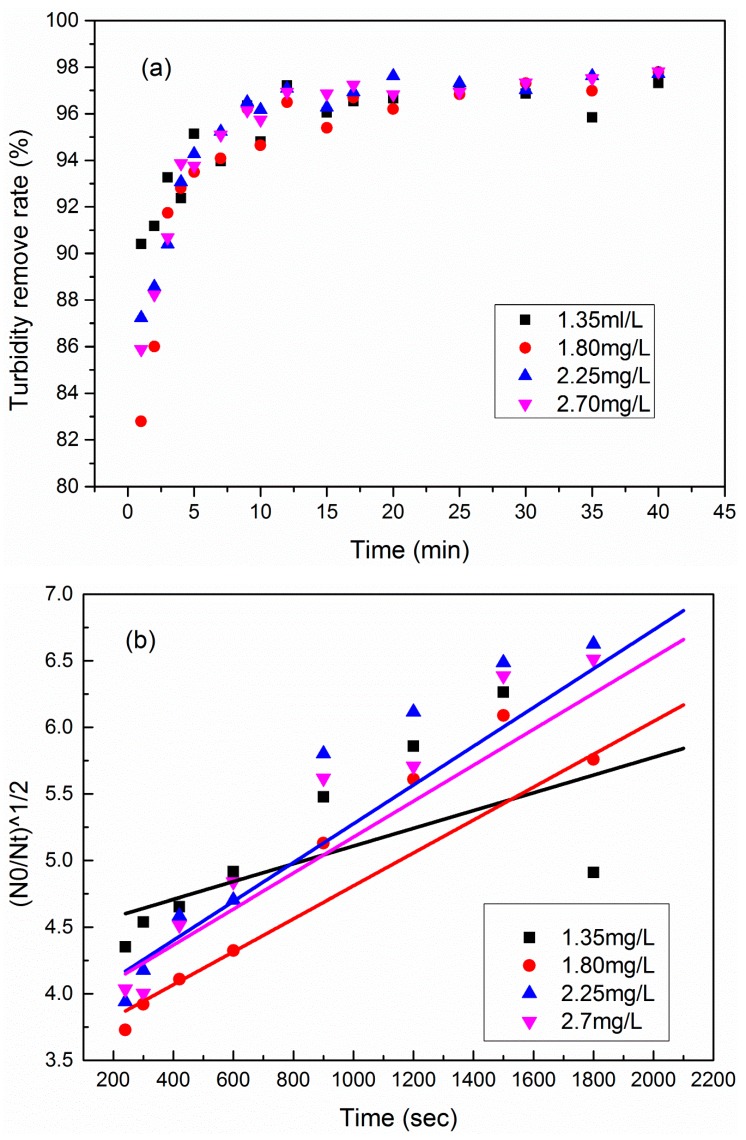
Turbidity removal rate with different PATC-PDDAAC dosage changes with flocculation time (**a**), and (N_0_/N_t_)^1/2^ changes with flocculation time (**b**).

Flocculation kinetics of PATC-PDMDAAC at various dosages were studied in this work to further understand the flocculation process. PATC-PDMDAAC with different amounts was added to the configured simulated water sample of humic acid-kaolin. The coagulation experiment was carried out at a fixed speed of 210 r/min to control different mixing times. The precipitation time was fixed at 30 min. The supernatants of different samples were collected for turbidity determination and kinetic analyzes. In this work, a particle collision flocculation kinetic model is adopted, and its expression according to the literature is as follows [35,38,39]:

(N_0_/N_t_)^1/2^ = 1 + 0.5kN_0_t (1)

where N_0_ is the initial content of the kaolin particle (measured by using initial turbidity in this work), N_t_ is the content of kaolin particles with respect to t (min) (measured by using residual turbidity), and k (s^−1^) is the kinetic constant of particle collision and aggregation. The experimental data demonstrate that the functional relationship between N_0_/N_t_ and t can be linearly fitted, and the fitting results are shown in Figure 13.

The kinetic constant (K) of particle aggregation can be calculated by the relation between (N_0_/N_t_)^1/2^ and time t according to the expression of particle collision flocculation kinetic model. The results of the relationship between (N_0_/N_t_)^1/2^ and time t under different dosage conditions are shown in Table 3. The table shows that the maximum value of KN_0_ is 27.8 × 10^−4^ s, and the corresponding dosage is the optimal one (i.e., 1.8 mg/L). This finding indicates that the collision between particles is effective under the optimal dosing condition. However, the corresponding KN_0_ value of high or low dosage decreases to a certain degree. This phenomenon is attributed to the water in the simulated water sample that is mainly in the form of negative charge, and the small amount of PATC-PDMDAAC added led to the small density of positive charge in the solution system. Consequently, few particles effectively collide, and the KN_0_ value is low. The increase in the coagulant dosage increases the number of positive charges in the solution system. Accordingly, the simulation of the adsorption of negative charge ions in water and the collision and contact between particles are facilitated to increase the K value. However, a certain degree of static repulsion is observed when the dosage is high. Such a phenomenon will reduce the connection point between effective particles and, thus, lead to the reduction of the K value.

### 3.6. Comparison of Actual Water Treatment

Table 4 shows the comparison of treatment effects of different coagulants on low-temperature and low-turbidity water. Liu et al. [40] studied the flocculation performance of PFM-PDMDAAC, and compared with the PAC and PFS, it was found that the composite flocculant has better treatment effect than traditional flocculant and the dosage of flocculant (3 mg/L) is only 50% of that of the traditional flocculant (6 mg/L). Zhang et al. [41] compared and analyzed the treatment effect of PAC and PAC-PDMDAAC on low-temperature and low-turbidity water. It was found that under the premise of ensuring the treatment effect, the dosage of compound flocculant PAC-PDMDAAC (2.66–2.53 mg/L) was reduced about 19.15–23.10% compared with PAC(3.17–3.11 mg/L), and the turbidity of the initial treatment was lower than 2NTU. Ma et al. [42] investigated the CBF and PAFC to treat raw water taken from Longhupao Reservoir in Heilongjiang Province for the removal of turbidity, it was found that the residual turbidity was 0.48 NTU (Nephelometric Turbidity Unit) by combining CBF (compound bioflocculant) of 2 mg/L and PAFC of 15 mg/L while the residual turbidity was 0.8 NTU when used PAFC only.

**Figure 14:**
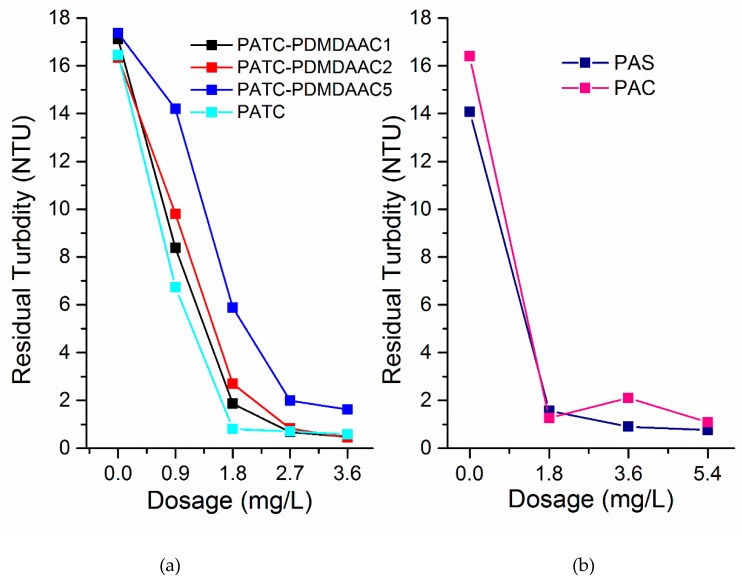
Comparison of effects of PATC-PDDAAC, PATC, PAS, and PAC on low-temperature and low-turbidity water in the Xiangjiang River. (**a**) the effect of PATC-PDMDAAC and PATC; (**b**) the effect of PAS and PAC.

In this research, PATC-PDMDAAC with different organic–inorganic ratios, PATC, and conventional flocculant PAC and PFS were compared for the treatment of low-temperature and low-turbidity water in the Xiangjiang River. The test surface water was taken from between the first and the third Xiangjiang bridges. Figure 14 shows the relationship between residual turbidities of Xiangjiang River water treated with different dosages of coagulant PATC-PDMDAAC (including PATC-PDMDAAC1, PATC-PDMDAAC2, and PATC-PDMDAAC5), PATC, PAC, and PAS (polyaluminium sulfate). The PATC-PDMDAAC and PATC advantages for treating the low turbidity and temperature water in Xiangjiang were clearly evident. The lowest residual turbidity of PATC-PDMDAAC2 treatment was 0.45 NTU at a dosage of 3.6 mg/L, while those of PATC, PAS, and PAC were 0.58 NTU, 0.76 NTU, and 1.09 NTU, respectively. The dosages of PAS and PAC were 5.4 mg/L. The composite flocculant is better than the conventional one for the treatment of Xiangjiang River water. The dosage was lower than the conventional flocculant while ensuring the treatment effect, and PATC-PDMDAAC can reduce the turbidity to a low level. However, the composite flocculant with a high organic–inorganic ratio was non-conducive to the treatment of low-temperature and low-turbidity water. This finding is consistent with the previous study on the treatment of organic and inorganic compounds to simulate low-temperature and low-turbidity water. This condition is attributed to the sweeping effect, which will occur early when the organic content of coagulant is particularly high, resulting in insufficient electric neutralization and adsorption bridging ability to reduce the treatment effect.

In order to better investigate the practical application of PATC-PDMDAAC, this study combined with the beaker test examined the materials used in the experimental process for brief cost estimation for reference in practical application. The specific contents are shown in Table 5. According to Table 5, theoretically, the raw materials cost about 185 RMB to prepare one ton of PATC-PDMDDACC (in liquid form).

## 4. Conclusions

A novel composite inorganic-organic coagulant (PATC-PDMDAAC) was prepared with PATC and PDMDAAC. The prepared composite coagulants were performed by FT-IR, XRD, TG-DSC, and other characterization analyses. The results showed that PATC and PDMDAAC were not simply physical mixtures, their bonds interacted and transformed, and the composite flocculant structure was relatively complex and stable.

The coagulant was used in the single-factor experiment of simulated low-temperature and low-turbidity water. The results showed the following optimal treatment conditions: organic/inorganic ratio of 0.15, optimal dosage of 1.8 mg/L, initial pH of treated water of nine, sedimentation time of 30 min, and stirring speed of 110 r/min. The analysis of ZP, 3D fluorescence, and kinetics showed that the mechanism of PATC-PDMDAAC for low-temperature and low-turbidity water treatment mainly relied on electric neutralization and adsorption bridging compared with PATC and PDMDAAC. The electric neutralization and adsorption bridging ability were improved. The collision between particles was effective under the optimal dosing condition. However, the dosing of PATC-PDMDAAC with high or low dosing was unfavorable to the treatment effect of low-temperature and low-turbidity water. PATC-PDMDAAC, PATC, and conventional coagulation treatment of low-temperature and low-turbidity water in the Xiangjiang River showed that the compound flocculant with an organic–inorganic ratio of 0.15 has the optimal treatment for the actual water, which can reduce the turbidity of the actual water sample to 0.45 NTU. The compound flocculant can not only ensure enhanced treatment effect but also reduce the dosage of the coagulant compared with the conventional one.

## Figures and Tables

**Table 1:** The characteristic of simulated and natural water.

Water	Characteristic	Unit	Value
Simulated water	Temperature	°C	5
	Turbidity	NTU	10
	pH	–	7.8 ± 0.2
	UV254	mg/L	10
	TOC	mg/L	4–8
Natural water (Xiangjiang River)	Temperature	°C	7.5 ± 0.3
	Turbidity	NTU	14–18
	pH	–	7.24 ± 0.2

**Table 2:** Fluorescence peak position and intensity comparison of water samples before and after coagulation under different pH conditions.

pH	The Dosage of PATC-PDMDAAC (mg/L)	EM (nm)	EX (nm)	Fluorescence Intensity
pH = 5.0	0	473	274	5558
	0.45	443	274	3069
	0.90	461	272	2822
	1.35	434	270	2990
	1.80	446	270	2655
	2.25	446	270	2589
pH = 7.0	0	485	270	7219
	0. 45	476	270	5504
	0.90	446	256	3199
	1.35	446	260	2802
	1.80	437	260	3079
	2.25	437	262	2843
pH = 9.0	0	476	272	7053
	0. 45	452	274	5603
	0.90	440	258	3360
	1.35	464	266	2532
	1.80	455	262	2856
	2.25	443	262	2717

**Table 3:** Simulation results of flocculation kinetics with different coagulant dosages.

Dosage	1.35 mg/L		1.80 mg/L		2.25 mg/L		2.70 mg/L	
	KN_0_ (× 10^−4^ s)	R^2^	KN_0_ (× 10^−4^ s)	R^2^	KN_0_ (× 10^−4^ s)	R^2^	KN_0_ (× 10^−4^ s)	R^2^
PATC-PDMDAAC	21.4	0.833	27.8	0.995	23.4	0.858	24.6	0.909

**Table 4:** Comparison of treatment effects of different coagulants on low-temperature and low-turbidity water.

Coagulants	The Characteristics of Water			Dosage mg/L	Removal Efficiency	Reference
	Initial Turbidity NTU	Temperature °C	pH			
PFM-PDMDAAC	17.4–21.5	7–9	7.15–7.32	3	95%	[40]
PAC				6	90%	
PFS				6	92%	
PAC-PDMDAAC	25–26	5–9	/	2.66–2.53	Below2 NTU	[41]
PAC				3.17–3.11		
CBF+PAFC	2.05	4.7	8.2	2 + 15	0.48 NTU	[42]
PAFC				20	0.8 NTU	

**Table 5:** The experimental cost estimate.

Material	Specification	Unit	Unit Price (RMB/t)	Consumption	Total (RMB/t)
AlCl_3_	Industrial grade	t	1550	0.0254	39
Na_2_SiO_3_	Industrial grade	t	2880	0.0159	46
TiCl_4_	Industrial grade	t	7000	0.0057	40
NaOH	Industrial grade	t	2600	0.0011	3
H_2_SO_4_	Industrial grade	t	850	0.0322	27
PDMDAAC	Industrial grade	t	8500	0.0036	30
PATC-PDMDAAC	–	–	–	–	185
